# Homeless mortality data from East London

**DOI:** 10.1080/17571472.2018.1458443

**Published:** 2018-04-05

**Authors:** Khalil Hassanally, Miqdad Asaria

**Affiliations:** a Health E1, The Greenhouse, London, UK; b Usher Institute, University of Edinburgh, Edinburgh, UK; c Centre for Health Economics, University of York, York, UK

**Keywords:** Inequality, mortality, housing, clinical audit

## Abstract

**Background:**

The rate of homeless mortality is known to be significantly below the national average, with mortality rates varying geographically. This study aims to look at the rates and causes of homeless mortality within East London.

**Question:**

To characterise homeless mortality of patients registered in two specialist homeless practices, between 2001 and 2016 in the London boroughs of Tower Hamlets and Hackney, by age at death and cause of death.

**Study Design:**

A retrospective study of general practice electronic patient records.

**Methods:**

Electronic patient records across two general practice surgeries specialising in care for the homeless in East London were examined and their mortality data extracted.

**Results:**

Two hundred and three deaths recorded in the two general practice surgeries were examined. The average age at death was 47 years, with the highest numbers of deaths being attributed to substance misuse, liver disease and cardiac-related deaths. Those dying of cardiac-related causes died at an average of 51, those dying of liver-related causes died at an average age of 49 years and those dying from substance misuse died at an average age of 38.

**Conclusions:**

Those dying of substance misuse-related causes died much younger than the average homeless patient did.

## Related LJPC papers

(2009) Royal College of General Practitioners Position Statement: Mental Health and Primary Care, London Journal of Primary Care, 2:1, 8–14, DOI: 10.1080/17571472.2009.11493235

## Why this matters to me

The homeless population have a life expectancy almost thirty years lower than the national average, a statistic that has remained sadly unchanged for over two decades. Since qualifying as a GP just under three years ago, I have worked predominantly within homeless primary care and am continually saddened by receiving death notification of patients, who seem to die needlessly young. When I worked as clinical lead for the Greenhouse practice (a homeless practice in Hackney), I often met with representatives from the council and the Greater London Authority to look at what interventions could be put into place to improve the health of the homeless within the local area. Frequently they would cite a lack of data as part of their problem in deciding what actions they could take, and whilst this paper by no means bridges that gap, hopefully it is a small step in addressing it. Working closely with the homeless population has also made it clear how important substance misuse services are, and how cuts in their provision are likely to affect this most vulnerable of demographics.

## Key Message

The age at death amongst London’s homeless population is much lower than that of the general population. Many of these early deaths that drive down this average are attributable to substance misuse.

## Introduction

In 2012, the homeless charity Crisis produced a report that looked into homeless mortality across the UK between the years 2001–2009. The findings were stark. The average age of death for men was 47 years whilst in women it was even lower at 44 years [1]. These figures have little changed over the past 25 years; in 1992 a working party of the Royal College of Physicians [2] also found the average age of death for homeless men to be 47 years. Regional variations do however exist; both Hewitt et al. [3] and Morrison [4] looking at mortality within Leicester (1989–2009) and Glasgow (2000–2005) respectively found an age of death closer to 41 years.

Drugs and alcohol abuse were important contributors to deaths in all these previous studies. In the Thomas report homeless people were found to be up to a nine-times more likely to die of an alcohol related condition and twenty times more likely to die from drugs than those in the general population [1]. Again, regional variations were found, London (which recorded almost a third of the deaths) was found to have the highest rates of cardiovascular deaths (almost a quarter of the deaths); London also had the lowest figure from deaths related to drugs, which accounted for 12.5% of total deaths [1].

## Methodology

This paper examines the deaths of over two hundred patients in the adjoining London boroughs of Tower Hamlets and Hackney who are registered in two specialist homeless practices. The data has been gathered by going through the electronic patient records of patients that have been marked as deceased between the years 2001–2016.

Where possible, causes of death have been taken from the death certificate. In some cases where the death certificate has been unavailable, the cause of death is inferred from coroners’ reports or discharge letters from the hospital.

The records were also screened for alcohol and drug use. Patients who were clearly marked as being in remission were not coded for substance misuse. Drug and alcohol use was not recorded for all patients (this was especially true when the patient died shortly after joining the practice or did not engage with the practice for long periods of time). Alcohol use tended to vary within the patient record (as patients tried to abstain or as their social circumstances worsened they drank more), and a modal value was used to represent the number of units drunk by a patient. Illicit substance use was not quantified in a consistent manner in the medical records. We therefore recorded substance abuse as a yes/no value capturing whether or not there was any record of substance abuse rather than the degree of substance abuse.

As well as looking at the cause of mortality, we also considered contributing factors and place of death.

## Results

**Figure 1.**
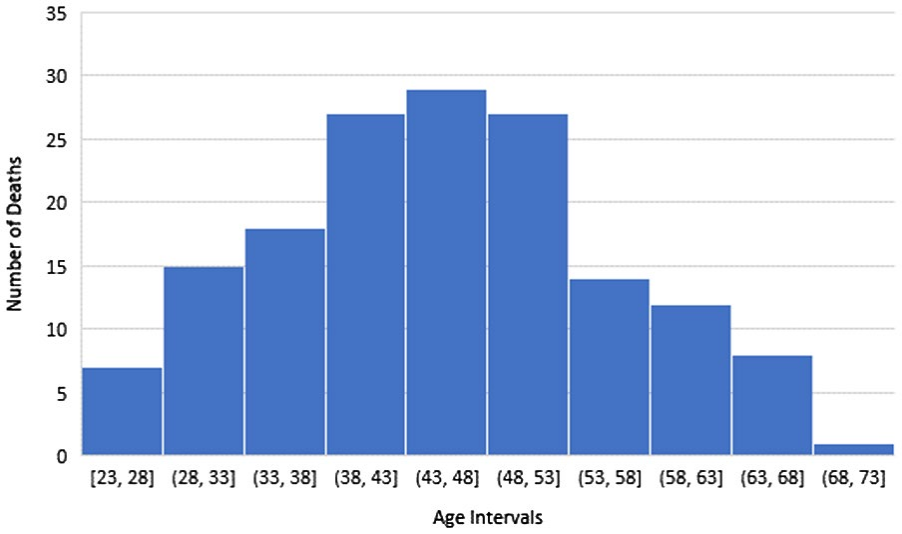
Number of deaths plotted against five yearly age intervals.

The average age at death across both the practices studied was 47 years, which is the same age as that discovered in the 2012 Crisis study [1]. Figure 1 shows the breakdown of number of deaths by age.

Of the 203 deaths observed in the study, the cause of death in 73 patients could not be determined from the medical records (from the death certificate, coroners’ reports and hospital notification) due to incomplete medical records. The causes of death of the remaining 130 patients are illustrated in the table below.

**Figure 2.**
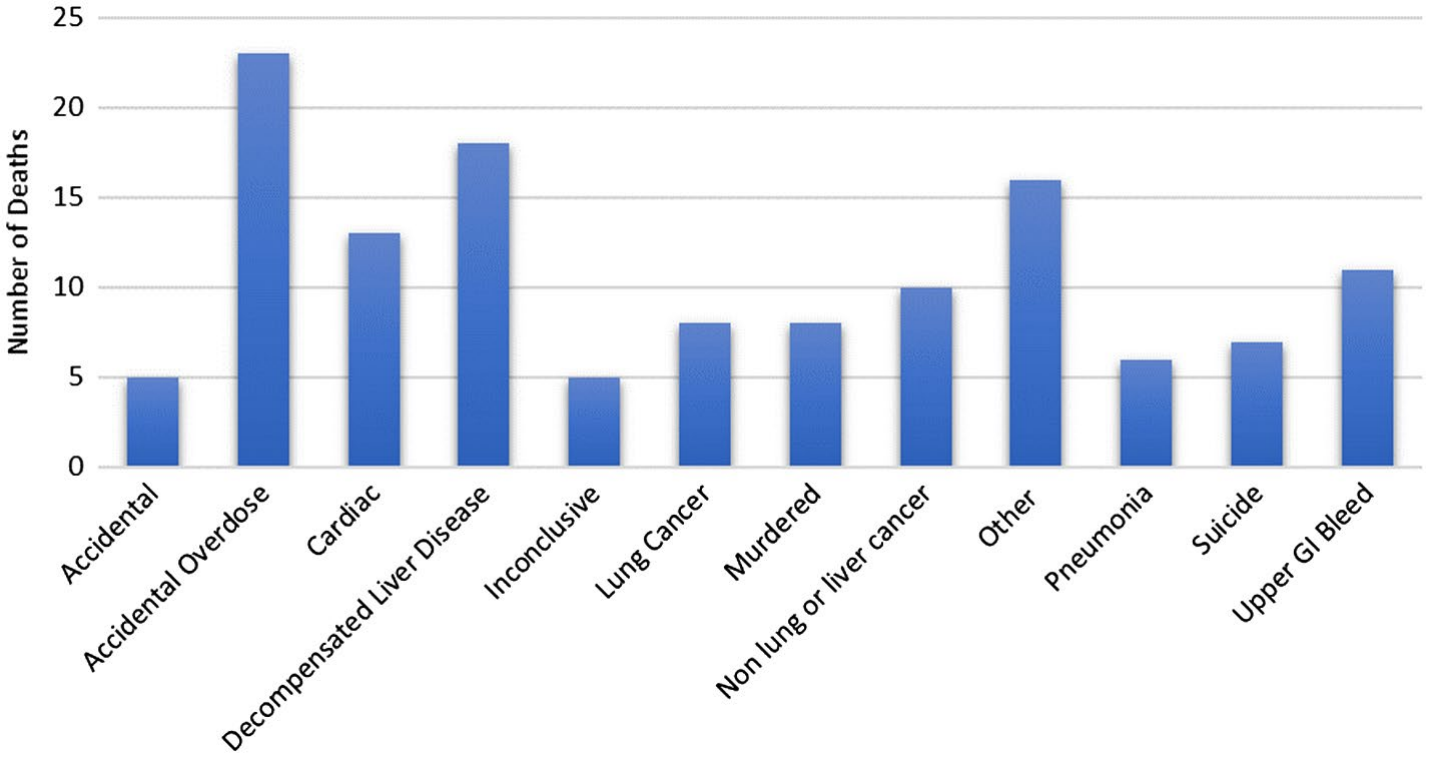
Analysis of the causes of mortality within the London Boroughs of Hackney and Tower Hamlets.

As can be seen in Figure 2, the largest share of deaths come from accidental overdose. Whilst decompensated liver disease and upper gastrointestinal bleeds are recorded separately they both have alcohol as a causative factor and if combined they would overshadow the deaths from overdose. Furthermore, it is difficult to know how many accidental overdoses were in fact suicide attempts. Cardiac causes of death made up 10% of deaths in our East London homeless population, as opposed to the 25% found by Thomas across the country [1]. Of the cardiac deaths, 61.5% were from an acute myocardial infarction.

The average age at death varied according to cause. Those dying of cardiac causes had died at an average of 51 years, whereas patients dying as a result of alcohol had an average age of mortality of 49 years, and those that died from overdose had an average age of death of 38 years.

Examining those patients that were recorded to have had alcohol related problems (49%), the average number of units drunk a week was 153 (ranging from 1 unit a week to 525 units a week with a median value of 140 units a week). Drug use has been recorded less consistently within the medical notes, however 43% of those homeless patients who died were noted to have been heroin users at some point during their lives (though this is likely an under-representation).

6.4% of the deaths occurred in the hospice, which is the same proportion of deaths that occur in a hospice setting within the non-homeless population [5]. At least eight of the deaths were of patients found in a decomposed state, their bodies having been found sometime after their deaths.

## Discussion

Whilst providing a useful snapshot of homeless deaths within East London this study is limited by the incomplete nature of the electronic medical records and the relatively small sample size. Additionally, whilst many homeless patients within Tower Hamlets and Hackney would have been registered at these specialist primary care services, some invariably will be registered elsewhere or not be registered at all. Assuming the latter group consists of those who have the more chaotic lives perhaps due to more troubled relationships with drugs and alcohol, then including these patients in the study would likely reduce the average age at death observed and further skew the causes of death towards those that are related to substance misuse. Similarly, not all the patients registered at the practices studied were actually homeless. It is therefore likely that average age at death of all homeless in East London is even lower than discovered in this study and the proportion of homeless dying of substance abuse is higher than in this study is even higher.

It is difficult to know how accurate a retrospective accounting of alcohol is. Invariably, consumption fluctuates according to circumstances, and where possible, a modal average was taken. Accurate associations between units of alcohol drunk and mortality are difficult to validate from a review of the medical notes, and more work needs to be done to clarify this.

This paper suggests that overall homeless mortality rates within Tower Hamlets and Hackney are in line with the national average expectations for homeless mortality. It additionally reinforces the fact that most deaths in homeless people are premature and from preventable causes. Given London’s unique position as having a number of different specialist primary care services for the homeless it would be useful to undertake further comparisons to see whether these results are consistent across the city. Further analyses would be useful of the health of the homeless that have access to health care compared with those who do not. Multi-morbidity is well recognised within the homeless population [6]; and the relationship between practice level risk factor management and cause of death is also an important area of future research.

Given emerging reports of rising mortality rates from heroin [7] and this study shows that those dying of overdoses tend to do so at a younger age, it is a concern that the average age at death will fall even more within the homeless population.

## Governance

Ethical approval obtained from East London Foundation Trust

## Disclosure statement

No potential conflict of interest was reported by the authors.
